# Drug administration errors among anaesthesia providers in South Africa: a cross-sectional descriptive study

**DOI:** 10.1186/s12871-024-02657-9

**Published:** 2024-08-03

**Authors:** René van Wyk, Ryan Alroy Davids

**Affiliations:** Department Anaesthesiology and Critical Care, University of Stellenbosch, Parow, Cape Town, 7500 South Africa

**Keywords:** Anaesthesia errors, Contributing factors, Drug administration errors, Medication errors, Reporting of drug errors

## Abstract

**Background:**

Drug administration errors (DAEs) in anaesthesia are common, the aetiology multifactorial and though mostly inconsequential, some lead to substantial harm. The extend of DAEs remain poorly quantified and effective implementation of prevention strategies sparse.

**Method:**

A cross-sectional descriptive study was conducted using a peer-reviewed survey questionnaire, circulated to 2217 anaesthetists via a national communication platform. The aim was to determine the self-reported frequency, nature, contributing factors and reporting patterns of DAEs among anaesthesia providers in South Africa.

**Results:**

Our cohort had a response rate was 18.9%, with 420 individuals populating the questionnaire. 92.5% of surveyed participants have made a DAE and 89.2% a near-miss. Incorrect route of administration, potentially resulting in serious harm, accounted for 8.2% (*n* = 23/*N* = 279) of these errors. DAEs mostly reported in cases involving adult patients (80.5%, *n* = 243/*N* = 302), receiving a general anaesthetic (71.8%, *n* = 216/*N* = 301), where the drug-administrator prepared the drugs themselves (78.7%, *n* = 218/*N* = 277), during normal daytime hours (69.9%, *n* = 202/*N* = 289) with good lightning conditions (93.0%, *n* = 265/*N* = 285). 26% (*n* = 80/*N* = 305) of DAEs involved ampoule misidentification, whilst syringe identification error reported in 51.6% (*n* = 150/*N* = 291) of cases. DAEs are often not reported (40.3%, *n* = 114/*N* = 283), with knowledge of correct reporting procedures lacking. 70.5% (*n* = 198/*N* = 281) of DAEs were never discussed with the patient.

**Conclusions:**

DAEs in anaesthesia remain prevalent. Known error traps continue to drive these incidents. Implementation of system based preventative strategies are paramount to guard against human error. Efforts should be made to encourage scrupulous reporting and training of anaesthesia providers, with the aim of rendering them proficient and resilient to handle these events.

**Supplementary Information:**

The online version contains supplementary material available at 10.1186/s12871-024-02657-9.

## Background

Drug administration errors (DAEs) in anaesthesia are common. From retrospective reviews and self-reported incident studies, the international incidence of drug administration errors in anaesthesia are reported to be around 1 in every 133–450 anaesthetics [1–4]. Data from prospective, observational studies report a much higher incidence of 1 error per every 20 perioperative medication administrations, translating to every second operation involving a medication error or adverse drug event [1, 5]. In a 2006 survey by Gordon and colleagues, 94% of South African anaesthetists reported that they have made at least one error and 22,6% reported at least four errors [6]. In a 2009 prospective study, Llewellyn and colleagues investigated academic facilities in South Africa and found a medication error incidence rate of 1 in every 274 anaesthetics performed [7]. 

Anaesthesiologists are required to order, dispense, administer, and monitor high-risk drugs while performing a variety of additional tasks in a complex work environment [1]. This process contains all necessary elements to create the perfect storm, surmounting in drug error, with varying degrees of harm and sequelae. [8]

Many anaesthesia-related DAEs are inconsequential, however, some lead to substantial or permanent injury and even death [9]. Literature reports incidence of morbidity and mortality of between 1 and 33% [2]. In fact, the risk of harm extends from the patient to the anaesthesia provider, the institution and medical profession as a whole – physical harm, psychological trauma, medico-legal implications, financial cost and loss reputation and public trust [1, 10, 11]. 

Despite recommended guidelines, which makes provision for prompt reporting of all medication related errors, DAEs in anaesthesia are notoriously difficult to study and vastly underreported [10–14]. 

Underreporting limits the capacity of healthcare workers and organisations to learn from these errors. Scrupulous reporting unveils the exact extent of DAEs with consequent heightened awareness contributing to improved patient safety [10]. 

This research aims to build and expand on current knowledge on DAEs in South Africa, specifically with regards to the self-reported frequency of a DAE, exploring the contributing factors, reporting patterns and anaesthetists’ perceptions of DAE reporting. Improved knowledge will raise awareness among anaesthetists and inspire culture change ultimately urging them to implement preventative measures to minimize the occurrence of future DAEs.

## Methods

### Study design and setting

A cross-sectional descriptive study was performed using a peer-reviewed survey questionnaire requesting respondents to self-report their experience with DAEs during anaesthesia. (Supplementary file 1). The questionnaire was distributed electronically via a national database. Weekly reminders were also distributed via the above system. Both the survey and reminders were aimed at anaesthesia providers who included specialist anaesthesiologists, trainees in anaesthesiology, diplomat anaesthetists, general practitioners, and/or medical officers, practicing anaesthesia in the private and/or public sector.

### Questionnaire

The survey questionnaire used in this study was developed by the authors and has not been published anywhere before. A thorough literature review was performed via PubMed and Google Scholar to inform the above research question looking at the most recent literature, but also reviewing landmark studies in the field.

The survey questionnaire was designed by the authors using Research Data and Electronic Capturing (REDcap) Consortium and was subjected to face validation [15, 16] utilizing experts in the field. All expert feedback received due consideration and were incorporated into the final survey. These experts were not financially compensated but suitably acknowledged in the publication.

The survey questionnaire included an introduction page, which pertained all necessary information as to the aim and intentions of the study and time estimate for completion of the survey. Participants completed the survey questionnaire on a strict voluntary and anonymous basis. Informed consent was accepted as completion of the survey.

The questionnaire consisted of five sections. The introductory section included two questions on demographics of the anaesthesia providers (qualification and years of experience) and five questions regarding the frequency and specific type of DAE or near-miss. Section 2 had 14 questions regarding the circumstances relating to the practitioner’s most memorable DAE. Section 3 consisted of eight questions regarding potential mitigating factors. Section 4 included six questions regarding the consequences of the DAE. Section 5 was about the reporting of DAE and consisted of nine questions.

Ethical approval was obtained from the Health and Research Ethics Committee of the University of Stellenbosch (ref. no. S22/03/049).

### Data analysis

The data was captured and stored on the REDcap system in an anonymized version. The data was analysed using Stata 17 software. Descriptive statistics were represented in graphical and numerical form. Percentages and frequencies were used, and cross-tabulations done [17–19]. 

## Results

### Demographics

At the time of the survey, there were 2217 anaesthesia providers on the national database. The response rate was 18.9%, with 420 individuals populating the questionnaire. Some respondents however, only populated certain areas of the questionnaire.

Most participating respondents were specialist anaesthetists (62.0%, *n* = 233/*N* = 376), with an average work experience of more than 10 years (Table 1).

**Table 1:** Data of respondents’ qualification and anaesthetic experience

Qualification		Anaesthetic experience (in years)					
		**< 2**	**3–5**	**6–10**	**11–15**	**> 15**	**Total**
MO/GP/DA*	n %	10 21.7	14 30.4	10 21.7	4 8.7	8 17.4	46 100.0
Registrar	n %	3 3.1	69 71.1	25 25.8	0 0.0	0 0.0	97 100.0
Specialist	n %	0 0.0	4 1.7	72 30.9	44 18.9	113 48.5	233 100.0
Total	n %	13 3.5	87 23.1	107 28.5	48 12.8	121 32.2	376 100.0

*MO: medical officer/GP: general practitioner/DA: diplomat anaesthetist

### Self-reported frequency, and type of DAE

92.5% (*n* = 356/*N* = 385) of surveyed participants have made a DAE and 89.2% (*n* = 345/*N* = 387) a near-miss (*any incident with the potential to become an error*, e.g.,* wrong drug drawn up but not given).*

For most of the anaesthesia providers, the occurrence of a DAE was an isolated event (83.1%, *n* = 314/*N* = 378). When asked about the frequency of DAEs, 58.2% (*n* = 224/*N* = 385) of respondents stated to have made less than 5 DAEs in the preceding year.

The majority of respondents selected incorrect route as an error type never encountered (66.9%); whilst most respondents selected omission, followed by substitution and incorrect dose, as the most frequently occurring events (Table 2).

Incorrect route of administration was reported in 23 of the 279 events, 8.2%. The most common intended site mentioned was intravenous, whilst the most frequent unintended sites included intravenous and neuraxial.

**Table 2:** Frequency of DAE according to error type

Type of DAE *			Frequency				
			**Never**	**Very seldom/seldom**	**Less/More frequent**	**Most frequent**	**Total**
Omission	n %		33 9.9	189 56.4	73 21.8	40 11.9	335 100
Substitution	n %		90 27.6	205 62.8	20 6.1	11 3.4	326 100
Repetition	n %		134 41.9	148 46.3	38 11.9	0 0.00	320 100
Incorrect dose	n %		66 19.7	193 57.6	69 20.6	7 2.1	335 100
Insertion	n %		121 37.1	171 52.5	31 9.5	3 0.9	326 100
Incorrect route	n %		281 84.1	51 15.3	2 0.6	0 0.00	334 100

*Type of DAE: Omission (drug not given, or given too late); Substitution (incorrect drug given); Repetition (additional dose of drug given); Incorrect dose (of drug given); Insertion (drug given which was not intended at that time or any stage); Incorrect route

### Nature of the DAE

**Fig. 1:**
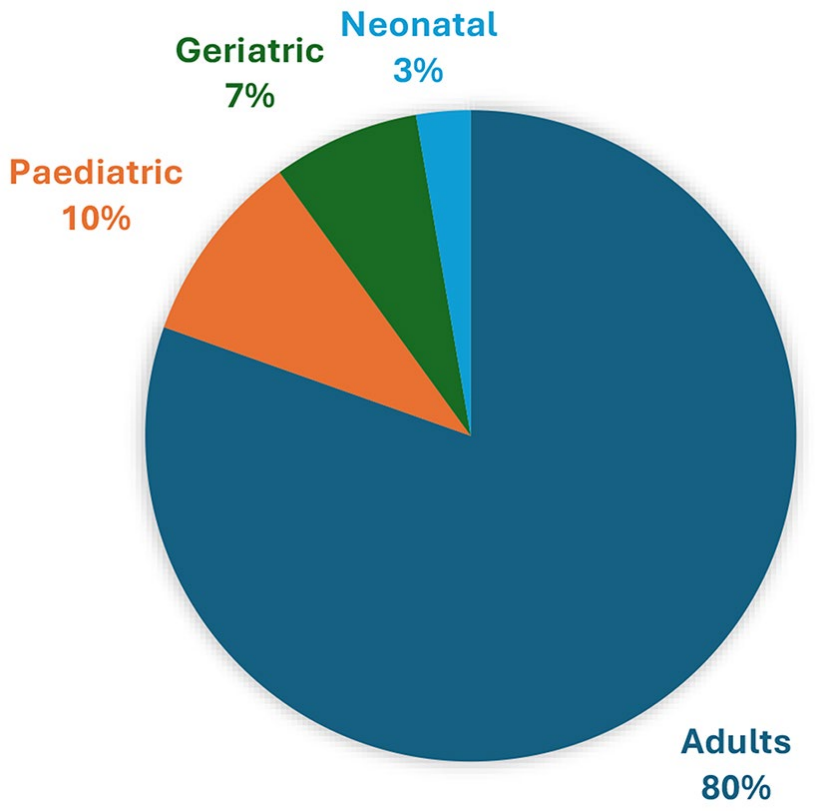
DAE per patient population

Most DAEs were made during the provision of anaesthesia to an adult population group (80.5%, *n* = 243/*N* = 302), followed by cases involving paediatric (9.6%, *n* = 29/*N* = 302), geriatric (7.3%, *n* = 22/*N* = 302) and neonatal (2.7%, *n* = 8/*N* = 302) patients (Fig. 1).

Respondents reported DAEs most often during cases involving patients from the American Society of Anaesthesiologists’ (ASA) physical status score II (43.6%, *n* = 129/*N* = 296), followed by ASA I (36.8%, *n* = 109/*N* = 296).

**Fig. 2:**
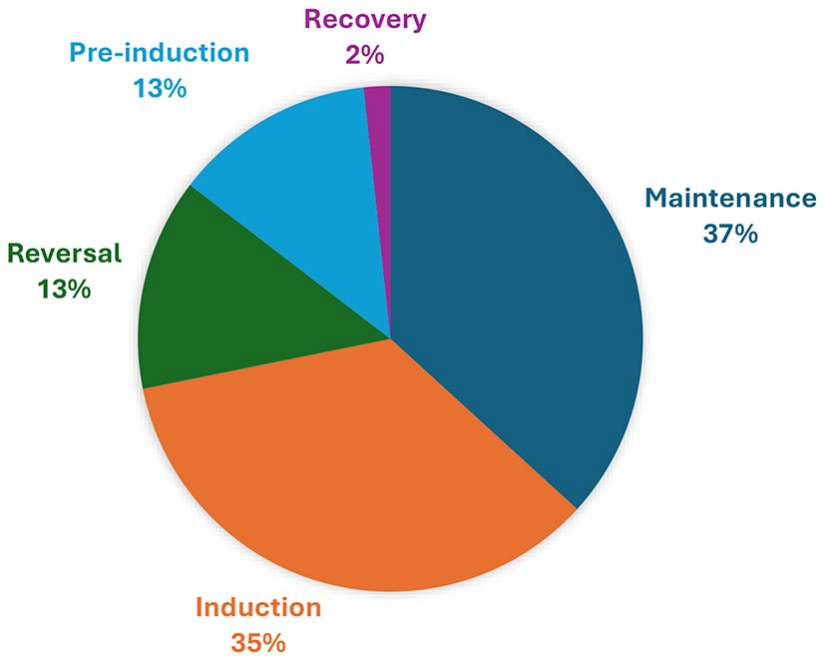
DAE per phase of anaesthesia

By and large, cases where a general anaesthetic were performed, accounted for the largest proportion of DAEs (71.8%) when compared to regional, combination or local only cases. The majority of DAEs was reported during the maintenance phase (36.8%, *n* = 111/*N* = 302) or during the induction of anaesthesia (35.1%, *n* = 106/*N* = 302)- as indicated in Fig. 2.

In this cohort, DAEs were reported most frequently during obstetric/gynaecological procedures (29.6%, *n* = 87/*N* = 294), followed closely by general surgery (24.5%, *n* = 72/*N* = 294) and orthopaedic surgery (16.7%, *n* = 49/*N* = 294). Less than 5% of DAEs were reported during cases of each of the remaining surgical specialities (including ear, nose, and throat surgery, paediatric surgery, urology, ophthalmology, cardiothoracic surgery, vascular surgery, maxilla-facial and oral surgery, neurosurgery, plastic surgery, colorectal, critical care, emergency medicine and speech therapy and audiology).

Most incidents were reported during elective cases (58.0%, *n* = 174/*N* = 300). Procedures with an average duration of 1–2 h had the highest occurrence of DAEs (45.3%, *n* = 136/*N* = 300), with the least amount of DAEs during cases of more than 4 h (3.3%, *n* = 10/*N* = 300).

We found that muscle relaxants (35.6%, *n* = 99/*N* = 278), vasoactive agents (16.9%, *n* = 47/*N* = 278) and opiates (13.0%, *n* = 36/*N* = 278) were the drugs most often involved in anaesthesia related DAE. In most of the DAEs, the drug administrator prepared the drug themself (78.7%, *n* = 218/*N* = 277).

### Contributory factors

A quarter (26,3%) of respondents stated ampoule misidentification as a contributory factor to their DAE. Similar looking ampoules 90.0% (*n* = 69/*N* = 77) and failing to read the label 71.8% (*n* = 56/*N* = 78,) accounted for the majority of these DAEs.

Syringe identification error was reported in 51.6% (*n* = 150/*N* = 291) of the DAEs. In 95.9% (*n* = 141/*N* = 151), of these cases, similar looking syringes or syringes of equal size were involved, whilst issues with labelling of the syringes (wrong, unclear, or confusing label) were mentioned as a contributory factor in two-thirds of cases.

Our study found the majority of DAEs were reported during normal day time hours (08:00–17:00) (69.9%, *n* = 202/*N* = 289) and with good lightning conditions (93.0%, *n* = 265/*N* = 285).

We found in most cases, only one anaesthesia provider was present at the time of the DAE (65.9%, *n* = 189/*N* = 287).

Fatigue was a contributory factor in only a third of incidents, mostly caused by tiredness (57.7%, *n* = 60/*N* = 104), a long case without a break (17,3%, *n* = 18/*N* = 104) and other, unspecified, causes (23.1%, *n* = 24/*N* = 104). Feeling rushed, pressured, or distracted accounted as a contributory factor in at least 50% of incidents and mentioned causes included the surgeon, the length of the list, another anaesthetist, being late or the telephone.

### Sequelae

In this research, we found that the anaesthesia provider realised they had made a DAE by a sudden change in the patient’s clinical condition (55.0%, *n* = 151/*N* = 275). Reviewing of the label of the ampoule or syringe (35.6%, *n* = 98/*N* = 275), and input from the anaesthetic assistant (5.1%, *n* = 14/*N* = 275) or from another clinician (4.4%, *n* = 12/*N* = 275) were other ways in which the anaesthesia provider became aware of the DAE.

The duration of the effects of the DAE was mostly short-lived, lasting only minutes in most cases (60.5%, *n* = 156/*N* = 258). The minority of respondents, less than 5.0% of cases, reported sequalae lasting days (1.6%, *n* = 4/*N* = 258) or being permanent (0.4%, *n* = 1/*N* = 258). Most respondents stated no therapeutic intervention was required after the DAE (60.1%, *n* = 169/*N* = 281).

Our research showed that a DAE mostly do not prolong the anaesthetic time 70.4% (*n* = 197/*N* = 280), however occasionally there is significant prolongation, 10.0% (*n* = 8/*N* = 81) stating more than 90 min.

Immediate effect of the DAE on the patient most notably resulted in apnoea (19.2%, *n* = 53/*N* = 276), sudden hyper- or hypotension (12.3%, *n* = 34/*N* = 276), sudden tachy- or bradycardia (8.0%, *n* = 22/*N* = 276) or pain (2.5%, *n* = 7/*N* = 276). Awareness was selected by less than 1% of the respondents. Anaphylaxis and cardiac arrest were each selected only once.

The outcome of the DAE was deemed by 88.3% (*n* = 249/*N* = 282) of respondents as having no clinical significance, while 10.6% (*n* = 30/*N* = 282) reported minor morbidity (temporary deviation in physiologic parameters) and 0.71% (*n* = 2/*N* = 282) major morbidity (derangement is permanent or leads to patient death). Only 1 respondent reported death as outcome for the DAE (0.4%, *n* = 1/*N* = 282).

### Reporting patterns

Our cohort demonstrated 40.3% (*n* = 114/*N* = 283) of respondents have never reported a DAE. Of the remaining 59.7%, informal reporting structures by far exceed formal reports, with only 5 respondents ever reporting to provincial or national level (3%, *n* = 5/*N* = 169).

At the time of completing the survey questionnaire, 54.5% of the anaesthesia providers were unaware of the reporting process at their facility (*n* = 153/*N* = 281) and 75.1% of respondents were unaware of national guidelines regarding reporting of DAE (*n* = 211/*N* = 281).

The population investigated attributed underreporting to the fact that the DAE was not regarded as serious enough to report (62.3%, *n* = 71/*N* = 114) or because the error held no sequelae for the patient (62.3%, *n* = 71/*N* = 114).

Unfamiliarity regarding reporting structures (26.3%, *n* = 30/*N* = 114); reluctance to engage in further paperwork (7.0%, *n* = 8/*N* = 114); and psychological safety issues including perceived lack of anonymity (3.5%, *n* = 4/*N* = 114); fear of blame(4.4%, *n* = 5/*N* = 114); fear of consequence(3.5%, *n* = 4/*N* = 114), embarrassment (14.0%, *n* = 16/*N* = 114), or legal action(7.9%, *n* = 9/*N* = 114) fuelled further underreporting.

The majority of DAEs were never discussed with the patient (70.5%, *n* = 198/*N* = 281).

Almost half of the respondents described their facility’s attitude towards DAE as supportive (49.6%, *n* = 138/*N* = 278), whilst others described it as indifferent (38.5%, *n* = 107/*N* = 278) or punitive (11.9%, *n* = 33/*N* = 278). 71.1% (*n* = 199/*N* = 280) of respondents stated that they felt safe to report a DAE.

The overwhelming majority of respondents stated that the outcomes of their reporting of the DAE was inconsequential (70.1% (*n* = 148/*N* = 211)). Only one third of respondents who had reported DAEs received constructive feedback.

Less than 50% of respondents deemed systemic safety measures at their facility to be adequate, while the rest regarded it as insufficient or even non-existent.

90.0% of responding anaesthesia providers report the DAE led to personal practice change.

## Discussion

Anaesthesiologists are required to independently execute the entire drug administration process while performing various other tasks in a dynamic work environment [1]. This process contains all necessary elements to create the perfect storm, surmounting in drug error, with varying degrees of harm and sequelae [8]. 

The World Health Organisation (WHO) reports that DAE accounts for more than half of all preventable harm in medical care globally, with an estimated annual cost of €4,5–21,8 billion in Europe [20, 21]. 

The self-reported frequency of DAEs, including near-miss, in this cohort is alarmingly high at 92.5% (*n* = 356/*N* = 385). This is concerning as the international incidence of DAEs in anaesthesia from retrospective reviews and self-reported incident studies are reported to be around 1 in every 133–450 anaesthetics [3, 4, 7, 22, 23]. It speaks to the great cognitive strain experienced by surveyed anaesthesiologists, with comparable findings locally [6, 7, 24]. 

Data from prospective, observational studies report a much higher incidence: 1 error per very 20 perioperative medication administrations, with every second operation involving a medication error or adverse drug event [5]. 

It highlights the demanding nature and brings into question the status quo where anaesthesiologists are required to order, dispense, administer, and monitor high-risk drugs while performing a variety of additional tasks in a complex work environment [1]. 

DAE encompass the misinterpretation of correctly written prescription, leading to administration of the wrong drug and/or wrong dose and/or drug at the wrong rate and/or wrong formulation or concentration and/or wrong route and/or wrong time and/or wrong patient [11]. A medication error only becomes an adverse drug event if the patient is harmed [11]. Pre-error (near-miss) is defined as any incident with the potential to become an error (e.g., wrong drug drawn up but not given) [5, 25]. 

DAEs occur because human error is unavoidable and the system by which medications are administered during anaesthesia is complex and tightly coupled with numerous latent factors predisposing to failure [26]. James Reason describe these latent conditions as resident pathogens, lurking in the system, waiting unnoticed until they are triggered by the right set of circumstances [1]. 

Our cohort identified various contributary factors to the occurrence of a DAE:

- Patient-related factors (adult patients, ASA I or II).
- Procedure-related factors (elective procedures, obstetric and gynaecological procedures, average procedure length 1–2 h).
- Anaesthesia-related factors (general anaesthetic, maintenance or induction phase, use of muscle relaxants, vasoactive agents, or opioids).
- Work-environment-related factors (drug-administrator prepared drugs, normal daytime hours, good lightning conditions, single anaesthesia provider present).

Although current literature states DAEs to occur more frequently in anaesthetic cases involving paediatric patients, our respondents indicated that most of their DAEs were made in the adult patient population group [27–29]. The majority of DAEs were reported during cases involving ASA I and II patients. Our findings agree with those by Hintong and colleagues, whereas Cooper and colleagues reported ASA III patients to have higher error rates, Nanji and colleagues found no association between error rates and ASA status [3, 5, 30]. The association, in our cohort, is believed to relate to cognitive load and increased vigilance during high stress procedures with potentially decreased vigilance during less complex anaesthetic procedures.

Error traps, described by Reason, are drug errors that occur and are reported repeatedly [11]. Such errors include omission, repetition, substitution, insertion, incorrect dose and incorrect route [4, 7, 12]. Substitution error occurs when a syringe or ampoule/vial swap result in incorrect drug administration instead of intended drug [3, 9]. These error traps, clearly remain an ongoing problem as represented in our cohort with most respondents having selected omission, followed by substitution and incorrect dose, as the most frequently occurring events [3, 4, 7, 11, 12]. 

DAEs resulting from incorrect route of administration, though infrequent, are known to be one of the error types associated with the greatest risk of harm [9, 25]. Unintended neuraxial drug administration, one of the most common sites involved in incorrect route of administration errors, is highly concerning as it poses severe immediate and longstanding adversity to the patient.

Most of the incidents in our study were reported during elective cases, which is supported by Hintong and colleagues’ findings [30]. In this cohort, obstetric/gynaecological procedures held the highest risk for DAE, followed closely by procedures in general surgery and orthopaedic surgery. The high-risk nature and unpredictability inherently associated with obstetric/gynaecological cases, together with the fact that these procedures are often performed on a high turnover list, pose further opportunity for mistakes. This differs from findings by Cooper and colleagues pointing towards cardiovascular/thoracic, colorectal, peripheral vascular, transplant and paediatric surgery as most often implicated specialities [3]. The majority of DAEs were reported during procedures with an average length of 1–2 h. With the least amount of DAEs during procedures of more than 4 h. This might be explained by the production pressure whilst doing a theatre list with a higher number of shorter patient procedures compared to a single, very long procedure. This contrasts with findings by Nanji and colleagues which concluded that longer procedures (> 6 h) had and those with 13 or more medication administrations higher event rates [5]. Differences in the methodology of these studies (prospective observational vs. self-reported descriptive) could account for these discrepancies.

DAEs incidence is proportional to the number of drugs administered over a given time period, making induction of general anaesthesia (GA) the highest risk for DAE. Additionally, this phase typically involves the integration of multiple tasks, including airway management, haemodynamic stability, and ventilation, whilst administrating various drugs in close succession [1]. This postulate was corroborated in this cohort. Surprisingly, a considerable number of DAEs were noted in the maintenance phase as well. These were thought to occur because of a decreased vigilance during this phase. Our findings are in keeping with those from Llewellyn, Orser, Hintong and Short [1, 7, 30, 31]. We found that muscle relaxants, vasoactive agents and opiates were the drugs most often involved in anaesthesia related DAEs. Given that this is echoed throughout existing literature, and notably that muscle relaxants, vasoactive agents and opioids were involved in patient harm more often than other drugs, increased vigilance and exceptional caution needs to apply when handling these drugs [1, 3–5, 7, 11, 12, 24, 25]. 

The majority of DAEs were reported during normal day time hours (08:00–17:00) and with good lightning conditions. This might speak to the fact that slips and lapses often occur while the anaesthetist is executing smooth, automated, and highly integrated tasks that has become routine activities to them and do not require conscious control or active problem solving [2]. Our study found that in most cases, the drug administrator prepared the drug themselves and, only one anaesthesia provider was present at the time of the DAE. This contrasts findings by Currie and colleagues [32], but might be explained by the inevitable multitasking, haste, production pressure, cognitive overload, inexperience, and individual fallibility whilst working alone in a well-recognised high-stress and busy environment with unpredictable scenarios, complex tasks, and interpersonal challenges [1, 9–11, 27, 32]. 

In the operating theatre, there are two mechanistic opportunities for error during the drug administration process. The first opportunity arises during the preparation of the medication syringe from the drug’s vial or ampoule. Choosing the wrong vial or the incorrect concentration are more likely when the drug containers look similar or are situated near each other in the anaesthesia drug tray. The second opportunity presents itself when the anaesthesia provider accidently chooses the wrong syringe, a process known as syringe swap or syringe substitution error [11, 27]. 

DAEs in anaesthesia are often preventable [2, 5, 9, 12, 25], with misidentification of syringes and drug ampoules being the most common cause of preventable mishaps [33]. 

A quarter (26.3%) of respondents stated ampoule misidentification as a contributory factor to their DAE. This is slightly less than the 36.9% reported by Llewellyn et al. [7]. Similar looking ampoules and failing to read the label accounted for the majority of these DAEs. This remains reason for great concern as reports of this mechanism of error, along with advocacy for its prevention is multi-fold throughout the literature. [7, 33]

Syringe identification error was reported in 51.6% of the DAEs. In 95.9% of these cases, similar looking syringes or syringes of equal size were involved, whilst issues with labelling of the syringes involved in two-thirds of cases.

This highlights the need for active and intentional intervention to prevent these errors. Evidence-based recommendations include every drug ampoule or syringe must be labelled legibly with the drug name, date and concentration; read and verify every vial, ampoule, syringe label before administration; use pre-filled syringes where possible; formal organisation and standardisation of drug trays and workspaces across all locations; checking of labels with a second person or a device (bar code reader); avoid similar packaging and presentation of drugs; reporting and reviewing DAEs. [9, 27, 34]Collaborative effort from individual anaesthesia providers, institutions and the pharmacological industry are required to successfully create change driving improved and sustainable patient safety.

Given the paucity of resources and emphasis on service delivery in low middle income countries like South Africa, it stands to reason that fatigue was found to be a contributory factor in 30% of incidents. Feeling rushed, pressured, or distracted accounted as contributory factor in 50%. Health care worker wellness and safe working hours enabling sound decision making, should be prioritised.

This is in agreeance with current literature pointing to the multifactorial nature of DAEs [3, 4, 11–13, 32, 34]. 

The WHO classifies morbidity caused by an incident using the following categories: [25]

1. No harm: when patient outcome is not symptomatic.
2. Minor and intermediate morbidity: when there is temporary deviation in physiologic parameters.
3. Major morbidity: when this derangement is permanent or leads to patient death.

Most respondents stated no therapeutic intervention was required after the DAE- minor morbidity with reversible harm (not requiring escalation of care) [3]. 

The outcome of the DAE was deemed by 88.3% of respondents as having no clinical significance, while 10.6% reported minor morbidity and 0.71% major morbidity. Only 1 respondent reported death as outcome. Our findings display the notion throughout literature that most DAEs were associated with minimal or no harm [1, 7, 9, 24]. Though the number of serious incidents is low, the incidence and preventability make it a noteworthy avenue for risk reduction [1, 2, 7, 9, 24]. 

DAEs in anaesthesia are vastly underreported. [10, 12–14]

Concerningly, this cohort demonstrated 40.3% of respondents have never reported a DAE.

Of the remaining 59.7% of the respondents stating that they have reported a DAE, informal reporting structures by far exceed formal reports, with only 5 respondents ever reporting to provincial or national level (3.0%, *n* = 5/*N* = 169). Reporting to managerial structures indicated by respondents in the minority of cases. Comparative data in the literature is sparse, highlighting the insurmountable nature of accurately quantifying the true scale and extent of the problem. The inability to measure impairs the opportunity for practitioners to learn and grow from these incidents and halts the implementation of adequate patient safety measures.

Our study demonstrated most practitioners were unfamiliar with formal reporting structures, at both facility and national level.

Underreporting could be due to reluctance to report, failure to recognise errors, the lack of understanding of how to report or what type of incidents should be reported. [5, 14]

The population investigated attributed underreporting to the fact that the DAE was not regarded as serious enough to report, or because the error held no sequelae for the patient. This is in keeping with findings by Catchpole and Burton [28, 35]. Unfamiliarity regarding reporting structures, reluctance to engage in further paperwork and psychological safety issues including perceived lack of anonymity, fear of blame, consequence, embarrassment, or legal action fuelled further underreporting in our cohort. Similar reasons are found in the literature [2, 12, 14, 28]. 

The majority of DAEs were never discussed with the patient, a finding similarly noted by Labuschagne and colleagues [24]. The anaesthesia provider’s proficiency and formal training in breaking bad news, navigating difficult conversations with patients along with their acceptance of susceptibility to making a DAE and acceptance of blame is highlighted by this finding.

Health care worker perception of the reporting process and the value thereof directly influences reporting rates [2, 14]. The overwhelming majority of respondents stated that the outcomes of their reporting of the DAE was inconsequential. Only one third of respondents who had reported DAEs received constructive feedback.

Psychological safety, which can be defined as a climate that instils in the members of the team a sense of confidence that they will not be rejected, punished, or embarrassed by the team when speaking up, is essential in ensuring a successful reporting system [2]. Such a system should be a system- orientated approach and together with professional leadership and organizational management aim to convert lessons learnt from incidents into systems improvements. [10, 12, 28]

Nearly 90.0% of responding anaesthesia providers report the DAE led to personal practice change.

Less than 50% of respondents deemed systemic safety measures at their facility to be adequate, while the rest regarded it as insufficient or even non-existent. This is concerning as research into human error clearly points to the fact that simply trying harder to avoid error, on its own is unlikely to be successful. Therefore, systems must be engineered to bypass human factors to prevent error from ever occurring [1, 2, 12, 27, 34]. 

As both the breadth of our pharmacological armamentarium and the complexity of anaesthesia care environments increase, individual utmost vigilance and institution-wide commitment and strategy for safer drug administration systems are the basis of a worthwhile and sustained improvement in anaesthesia medication safety [1, 2]. 

## Conclusion

DAEs in anaesthesia remain prevalent. Known error traps continue to drive these incidents. Implementation of system based preventative strategies are paramount to guard against human error. Efforts should be made to encourage scrupulous reporting and training of anaesthesia providers, with the aim of rendering them proficient and resilient to manage these events.

### Limitations

Self-reported surveys are potentially inaccurate with regards to the reported error rates, because of the difference between the likelihood of a practitioner to report a DAE versus the actual frequency thereof. Unnoticed errors do not get reported and the definition of error is subjective. The response rate was only 18.9% and therefore no generalisation can be made to South African anaesthesia providers as a whole and the results pertain to the responders only.

## Electronic supplementary material

Below is the link to the electronic supplementary material.

Supplementary Material 1

## Data Availability

The datasets used an/or analysed during the current study are available from the corresponding author upon reasonable request.

## Abbreviations

ASA: American Society of Anaesthesiologists
DA: Diplomat Anaesthetist
DAE: Drug Administration Error
GA: General Anaesthetic
GP: General Practitioner
MO: Medical Officer
ODAM: Order, Dispense, Administer and Monitor
REDcap: Research Data and Electronic capturing
WHO: World Health Organisation
