# Supplementary material for: Drug administration errors among anaesthesia providers in South Africa: a cross-sectional descriptive study
Source: BMC Anesthesiol. 2024 Aug 3;24:270. doi: 10.1186/s12871-024-02657-9 (PMC11297762; doi:10.1186/s12871-024-02657-9)
Supplement: Supplementary file 1 — Supplementary Material 1 [file 12871_2024_2657_MOESM1_ESM.pdf]

What is the incidence, contributing factors and reporting patterns of drug administration errors in South Africa?

Dear Colleague,

Thank you for your participation in this MMED Research Project titled:

“What is the incidence, contributing factors and reporting patterns of drug administration errors in South Africa?”

By completing this online survey questionnaire, you will help us in collecting pertinent information and ultimately improve patient safety.

Participation is entirely voluntary and completely anonymous. No personal identifiers will be recorded. Participants reserve the right to withdraw from this project at any stage.

This survey is user-friendly and will take 10-15 minutes to complete.

On completion of the survey, you will have the opportunity to enter the lucky draw.

The winner will be able to select a prize of their choice:

SASA Congress 2023 paid in fullTravelstart voucher (valued at R2500)Leather backpack (valued at R2500)Follow this link to start the survey: xxxxxxxxxxxxxxxxxx

Regards,

Dr René van Wyk

Email: rene.potg@gmail.com

1)	What is your qualification?	◯ Medical officer/General practitioner<br>◯ Diplomat anaesthetist<br>◯ Registrar<br>◯ Specialist anaesthetist
2)	Please state your anaesthetic experience (in years):	◯ < 2<br>◯ 3-5<br>◯ 6-10<br>◯ 11-15<br>◯ >15
3)	Have you ever made a drug administration error (DAE)?	◯ Yes<br>◯ No
4)	Have you ever had a near-miss? (any incident with the potential to become an error e.g. wrong drug drawn up but not given)	◯ Yes<br>◯ No
5)	How many DAE have you made in the last year?	◯ 0<br>◯ < 5<br>◯ 6-10<br>◯ 11-20<br>◯ >20

6) How often do you made a DAE?

- ◯ Daily
- ◯ Once every week
- ◯ Once every two weeks
- ◯ Monthly
- ◯ Yearly
- ◯ Isolated events

What type of DAE do you make most frequently?

Please score each error type from the least to the most frequent using numbers 1-5:
1: never, 2: very seldom, 3: seldom, 4: less frequent, 5: more frequent

		1	2	3	4	5
7)	Omission: drug not given, or given too late	◯	◯	◯	◯	◯
8)	Substitution: incorrect drug given	◯	◯	◯	◯	◯
9)	Repetition: additional dose of drug given	◯	◯	◯	◯	◯
10)	Incorrect dose of drug given	◯	◯	◯	◯	◯
11)	Insertion: drug given which was not intended at that time or any stage	◯	◯	◯	◯	◯
12)	Incorrect route	◯	◯	◯	◯	◯

13) Recalling your most memorable drug error

What was the type of surgery/procedure? ________________________________

14) What was the patient's age?
◯ Neonate ◯ Paediatric
◯ Adult ◯ Elderly

15) What was the surgical speciality?
◯ Cardiac & thoracic surgery
◯ Cardiology ◯ Colorectal
◯ Critical care ◯ nose and throat surgery
◯ Emergency medicine
◯ General surgery ◯ Gynaecology & obstetrics ◯ Maxilla-facial and oral surgery
◯ Neurosurgery ◯ Oncology
◯ Ophthalmology ◯ Orthopaedic surgery
◯ Paediatric cardiology
◯ Paediatric surgery ◯ Plastic surgery
◯ Psychiatry ◯ Radiology
◯ Speech therapy and audiology
◯ Transplant surgery ◯ Urology
◯ Vascular surgery

16) What was the ASA status of the patient?
◯ I ◯ II ◯ III ◯ IV
◯ V

17) Emergency or elective case
◯ Emergency ◯ Elective

18) Type of anaesthesia
◯ General ◯ Regional
◯ Local ◯ Combination

19) Length of procedure
◯ < 1 hr ◯ 1-2 hrs ◯ 2-4 hrs
◯ > 4 hrs

20) Phase of anaesthetic (when did the error occur)?
◯ Pre-induction ◯ Induction
◯ Maintenance ◯ Reversal
◯ Recovery

21) Was the error due to drug ampoule misidentification? ◯ Yes ◯ No

If due to drug ampoule misidentification error:

		yes	no
22)	Did you read the label of the ampoule prior to selecting the ampoule?	◯	◯
23)	Was the ampoules similar looking?	◯	◯

24) Was the error due to a syringe identification error? ◯ Yes ◯ No

If due to a syringe error:

	Yes	No
25) Were the syringes similar looking or of equal size?	○	○

If due to syringe error:

		wrong	unclear	confusing	other
26)	Was the labelling of the syringe	◯	◯	◯	◯

27) What was the specific drug involved (select drug class)?

◯ Muscle relaxant ◯ Antibiotic
◯ Opiate ◯ Sedative
◯ Vasoactive agent ◯ Inhalational agent
◯ Local anaesthetic ◯ Intravenous induction agent ◯ Anticholinergic agent
◯ Drug altering haemostasis

28) Please name the specific agent.

____

29) Did the drug administrator draw up the drug? ◯ Yes ◯ No

30) Was the DAE a result of incorrect route of administration? ◯ Yes ◯ No

If due to incorrect route of administration, what was the intended vs administered site:

		intravenous	intramuscular	neuraxial	regional	other
31)	Intended site	◯	◯	◯	◯	◯
32)	Administered site	◯	◯	◯	◯	◯

---

33) With regards to your most memorable DAE consider potential mitigating factors:

Time of day/night incident occurred

◯ 08:00-17:00 ◯ 17:01-00:00
◯ 00:01-07:59

---

34) How many anaesthesia providers present at the time of the incident?

◯ 1 ◯ 2 ◯ >2

---

35) How long have you been on duty when the DAE occurred (estimated in hours)?

____

---

36) How long since you last slept (estimated in hours)?

____

---

37) Was fatigue a factor?

◯ Yes ◯ No

---

38) If yes, provide most likely reason for fatigue:

◯ Tiredness ◯ Boredom
◯ Long case without a break
◯ Other

---

39) Lighting conditions

◯ Good ◯ Poor

---

40) Was the incident contributed to by you feeling rushed or pressured?

◯ Yes ◯ No

---

41) If yes, please select what made you feel rushed or pressured:

◯ Surgeon ◯ Another anaesthetist
◯ Length of the list ◯ Telephone
◯ Being late ◯ Other

---

42) Where you distracted?

◯ Yes ◯ No

---

43) If yes, please state the most likely reason for the distraction:

____

---

44) Consequences of the DAE:

What drew your attention to the error:

◯ Clinical effects ◯ Label
◯ Anaesthetic assistant
◯ Another clinician

---

45) What was the immediate effect of the DAE?

◯ Sudden hyper-/hypotension
◯ Sudden tachy-/bradycardia
◯ Apnoea ◯ Anaphylaxis
◯ Cardiac arrest ◯ Awareness
◯ Pain ◯ Other

---

46) If other, please specify:

____

47)	What was the duration of the effect of the DAE?	◯ Seconds ◯ Minutes<br>◯ Hours ◯ Days ◯ Weeks<br>◯ Permanent
48)	Was therapeutic intervention necessary?	◯ Yes ◯ No
49)	Did it prolong the anaesthetic?	◯ Yes ◯ No
50)	If yes, by how long?	◯ < 30 min ◯ 30-60 min<br>◯ 60-90 min ◯ > 90 min
51)	What was the final outcome of the DAE?	◯ Not of clinical significance<br>◯ Minor morbidity ◯ Major morbidity<br>◯ Death
52)	Attitudes towards reporting:<br><br>Have you ever reported a DAE?	◯ Yes ◯ No
53)	If yes, how did you report the DAE?	☐ To a colleague ☐ To your senior<br>☐ To the matron ☐ To theatre management ☐ To hospital management<br>☐ Provincially ☐ Nationally
54)	If unreported, what was the most likely reason?	☐ Did not regard error as serious enough to report ☐ No sequelae for the patient<br>☐ Did not have time for extra paperwork<br>☐ Not aware of reporting structures<br>☐ Lack of anonymity ☐ Fear of blame<br>☐ Fear of consequence<br>☐ Fear of embarrassment<br>☐ Fear of legal action ☐ Other
55)	If other, please state:	______________________________
56)	Did you discuss the DAE with the patient?	◯ Yes ◯ No
57)	What was the end-result of you reporting the error?	◯ Nothing ◯ Disciplinary action<br>◯ Medico-legal action ◯ Constructive feedback
58)	Do you regard systematic safety measures at your facility to be:	◯ Adequate ◯ Insufficient<br>◯ Non-existent
59)	Are you aware of the reporting process at your facility?	◯ Yes ◯ No
60)	Are you aware of national guidelines regarding reporting of DAE?	◯ Yes ◯ No
61)	How would you describe your facility's attitude towards DAE?	◯ Indifferent ◯ Punitive<br>◯ Supportive
62)	Do you feel safe to report DAE?	◯ Yes ◯ No

63) Did your DAE lead to personal practice change? ◯ Yes ◯ No
